# Genetically Proxied SGLT2 Inhibition and Risk of Epilepsy: A Mediation Mendelian Randomization Study of Circulating Metabolites

**DOI:** 10.3390/biomedicines14102406

**Published:** 2026-10-21

**Authors:** Mei Chang, Jiaxuan Li, Feng Gu, Xin Wu, Bingyi Song, Zhouqing Chen, Zhong Wang

**Affiliations:** Department of Neurosurgery & Brain and Nerve Research Laboratory, The First Affiliated Hospital of Soochow University, Suzhou 215006, China

**Keywords:** SGLT2 inhibition, epilepsy, diabetes, pharmacological targets, Mendelian randomization analysis

## Abstract

**Background:** Epilepsy is a chronic neurological disorder that can significantly impair quality of life. Previous studies have demonstrated the positive effects of sodium–glucose co-transporter 2 (SGLT2) inhibitors on various systems, including the nervous system. SGLT2 inhibitors have been shown to reduce seizure activity in pentylenetetrazol-induced epilepsy models in rats. Recent study results showed that glucose-lowering drugs including SGLT2 inhibitors reduced risk of seizures and epilepsy. **Methods:** Genetic variants of SGLT2 inhibition were selected as exposure instrumental variables (IVs) and used type 2 diabetes (T2DM) as validation. Two-sample Mendelian Randomization (MR) analysis was conducted to explore the causal relationship between SGLT2 inhibition and epilepsy. In addition, we performed a two-step MR to evaluate whether possible circulating metabolites mediate the effects of SGLT2 inhibition on epilepsy. **Results:** The two-sample MR analysis showed that genetically proxied SGLT2 inhibition was associated with lower odds of epilepsy under MR assumptions [IVW: OR = 0.30 (95% CI: 0.13, 0.71), *p* = 0.0064]. Exploratory two-step analyses identified 3-methylglutaconate and N-acetylleucine as candidate metabolic signals; however, neither signal remained statistically significant after Benjamini–Hochberg correction. **Conclusion:** Our study suggests that SGLT2 is associated with a reduced risk of epilepsy, and the underlying mechanism may be the potential antioxidant and anti-inflammatory effects of SGLT2. In addition, circulating 3-methylglutaconate and N-acetylleucine should therefore be regarded as candidate metabolic signals rather than established mediators.

## 1. Introduction

Epilepsy is a chronic neurological condition affecting an estimated 70 million individuals worldwide; recurrent seizures, psychiatric and mood comorbidities, cognitive impairment, and adverse treatment effects collectively impose a substantial burden on quality of life [1]. Current anti-seizure medications (ASMs) fail to provide adequate seizure control in approximately 30% of patients [2], underscoring the need to evaluate alternative pharmacological targets for epilepsy. Sodium–glucose cotransporter 2 (SGLT2) inhibitors lower glycaemia by blocking SGLT2-mediated glucose reabsorption at the apical membrane of renal proximal tubular cells. Consequently, less glucose is reclaimed by the kidney and more is excreted in urine, thereby reducing circulating glucose concentrations [3]. Evidence accumulated beyond diabetology indicates that the effects of SGLT2 inhibition extend to several organ systems, including the nervous system. Among patients with type 2 diabetes mellitus (T2DM) and acute myocardial infarction (AMI), exposure to an SGLT2 inhibitor has been associated with a lower inflammatory burden and a smaller infarct size. Experimental work in nondiabetic, doxorubicin-treated mice has likewise shown improved cardiac function accompanied by attenuation of ferroptosis, fibrosis, apoptosis, and inflammation. These benefits appear to involve NOD-like receptor family pyrin domain-containing 3 (NLRP3) inflammasome signaling and MyD88-related pathways [4]. Together, these observations broaden the evidence for cardio protection by SGLT2 inhibitors in coronary artery disease [5]. Renal benefits have also been attributed to reductions in glomerular capillary pressure and hyperfiltration, which relieve mechanical strain on the filtration barrier, diminish albuminuria, and reduce the oxygen required for tubular reabsorption [6]. Thus, the therapeutic consequences of SGLT2 inhibition encompass myocardial and renal protection in addition to glycaemic control. Emerging research suggests that its relevance to neurological disorders may reflect neuroprotective actions. Shared pathways and biomarkers between SGLT2 inhibition and conditions such as autism spectrum disorder (ASD) have also prompted consideration of these agents in neurodegenerative and related disorders. Collectively, these findings support biological effects of SGLT2 inhibitors that extend beyond metabolic disease [7]. Experimental evidence further points to an anti-inflammatory action of this drug class. In macrophages, for example, SGLT2 inhibition suppresses activation of the NLRP3 inflammasome [8,9]. Antioxidant activity represents another proposed component of SGLT2-mediated protection. Reported effects include lower free-radical production and enhancement of endogenous antioxidant defenses, including glutathione (GSH) and superoxide dismutase (SOD). Some SGLT2 inhibitors may also penetrate the blood–brain barrier (BBB), potentially enabling direct or indirect protection within the central nervous system [10]. By limiting oxidative injury and reinforcing antioxidant capacity, SGLT2 inhibition could therefore be relevant to neurological disorders characterized by redox imbalance. Consistent with this possibility, favorable effects have been described in experimental models of ischemic stroke, Alzheimer’s disease (AD), Parkinson’s disease (PD), and epilepsy [7,11].

In rodent models of epilepsy induced by pentylenetetrazol (PTZ), SGLT2 inhibitors have also reduced seizure activity. Two mechanisms have been proposed for this anticonvulsant action: restricting glucose availability and decreasing sodium transport across neuronal membranes [12]. These observations raise the possibility that SGLT2 inhibition could have therapeutic relevance in epilepsy. A recent meta-analysis similarly reported a lower incidence of seizures and epilepsy among users of newer glucose-lowering therapies, including SGLT2 inhibitors [13].

Mendelian randomization (MR) uses inherited genetic variants as instrumental variables to estimate associations that may inform causal inference under specific assumptions. Here, genetic colocalization was incorporated when selecting instruments linked to messenger RNA expression of the pharmacological target [14,15]. This framework permits assessment of whether genetically proxied perturbation of SGLT2-related biology is associated with T2DM and epilepsy under MR assumptions. Existing studies have considered relationships between glucose regulation and epilepsy, but their designs and the drug classes evaluated leave important uncertainties. A systematic MR investigation integrating genetically proxied SGLT2 inhibition, metabolomic traits, and epilepsy is therefore warranted. We performed two-sample MR followed by two-step MR to test whether circulating metabolites or metabolite ratios may lie on the pathway between genetically proxied SGLT2 inhibition and epilepsy risk. This analysis was intended to explore metabolic pathways that may be associated with the relationship between genetically proxied SGLT2 inhibition and epilepsy.

## 2. Methods

### 2.1. Study Design

**Figure 1:**
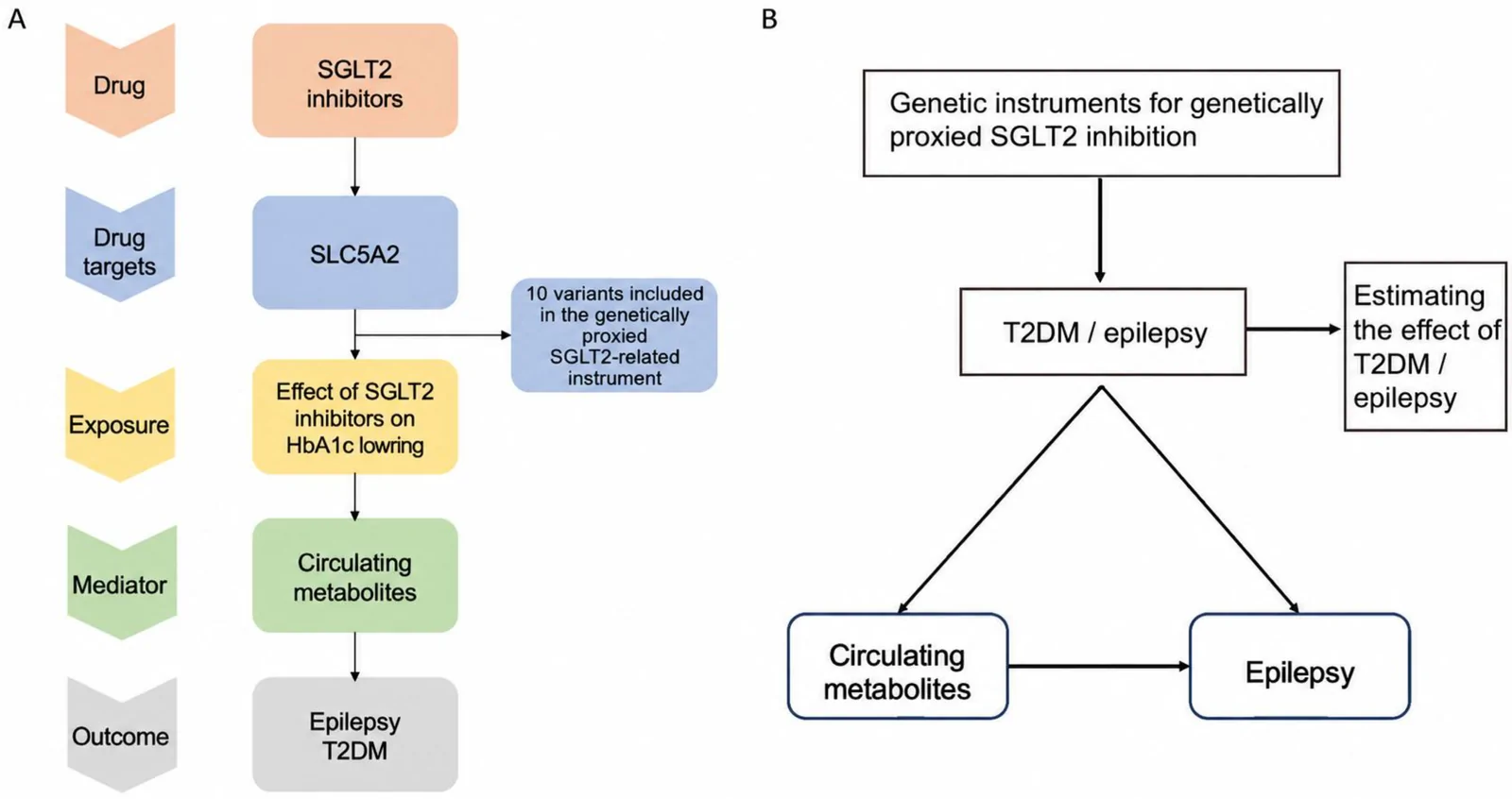
Overview of the study design. (**A**) Conceptual framework linking SGLT2 inhibition, the drug target SLC5A2, HbA1c lowering, circulating metabolites, and disease outcomes, with 10 genetic variants included in the instrument for genetically proxied SGLT2 inhibition. (**B**) Analytical workflow comprising two-sample Mendelian randomization to assess the associations of genetically proxied SGLT2 inhibition with T2DM and epilepsy, followed by exploratory two-step Mendelian randomization to investigate circulating metabolites as potential intermediates in the association with epilepsy. SGLT2, sodium–glucose cotransporter 2; SLC5A2, solute carrier family 5 member 2; HbA1c, glycated hemoglobin; T2DM, type 2 diabetes mellitus.

We first selected genetic variants that proxy SGLT2 inhibition as instrumental variables (IVs) and assessed instrument validity using T2DM as a positive-control outcome. Epilepsy was then analyzed as the primary outcome in a two-sample MR framework to estimate the association with genetically proxied SGLT2 inhibition. A two-step MR analysis was additionally undertaken to explore whether circulating metabolites might lie on the pathway between genetically proxied SGLT2 inhibition and epilepsy (Figure 1). Reporting followed the Strengthening the Reporting of Observational Studies in Epidemiology Using Mendelian Randomization (STROBE-MR) guideline [16,17], and the completed STROBE-MR checklist is provided in the Supplementary Materials. The validity of MR inference relies on three core instrumental-variable assumptions: (1) relevance, whereby the genetic instruments are associated with the exposure; (2) independence, whereby the instruments are independent of confounders of the exposure–outcome relationship; and (3) exclusion restriction, whereby the instruments influence the outcome only through the exposure and not through alternative pathways [18]. These assumptions were evaluated where possible using instrument-strength statistics, LD clumping, MR-Egger regression, MR-PRESSO, and heterogeneity analyses.

### 2.2. Selection and Validation of Genetic Instruments for Genetically Proxied SGLT2 Inhibition

Genome-wide association study (GWAS) resources from the Genotype-Tissue Expression (GTEx) project [18] and the eQTLGen Consortium [19] were queried for variants associated with SLC5A2 expression. For each SLC5A2 variant, its association with glycated hemoglobin (HbA1c) was examined, and variants associated with HbA1c at *p* < 1 × 10^−4^ were retained [19]. Summary statistics for HbA1c were obtained from the UK Biobank (*n* = 344,182). Colocalization analysis was used to evaluate whether SLC5A2 expression and HbA1c were driven by a shared causal variant [20]. A prespecified posterior-probability threshold of >70% was used as a screening criterion during instrument construction. Because complete locus-level diagnostic outputs were not available for the downstream MR analysis, the colocalization step was not interpreted as independent validation of the subsequent epilepsy association. To minimize correlation among the genetic instruments, LD clumping was performed using the European ancestry reference panel from the 1000 Genomes Project, applying a prespecified threshold of r^2^ < 0.001 within a 10,000-kb window [21]. Variants retained after this procedure are therefore referred to as LD-clumped variants rather than assumed to be completely independent. A positive-control MR analysis with T2DM was additionally performed to assess consistency with the expected glycaemic direction.

### 2.3. Genetic Instruments for Circulating Metabolites

Summary-level GWAS data for 1091 circulating metabolites and 309 metabolite ratios were derived from 8299 participants in the Canadian Longitudinal Study on Aging (CLSA) cohort [22]. Because few variants reached conventional genome-wide significance for some metabolites, instruments were selected using a relaxed association threshold (*p* < 1 × 10^−5^) and clumped at r^2^ < 0.1 within a 1000-kb window.

### 2.4. Outcome Data Source

GWAS summary statistics for epilepsy and type 2 diabetes mellitus (T2DM) were obtained through the IEU OpenGWAS resource and corresponded to the European-ancestry GWAS meta-analyses reported by Sakaue et al. The epilepsy dataset (GWAS ID: ebi-a-GCST90018840) included 4382 cases and 453,928 controls (total *n* = 458,310), whereas the T2DM dataset (GWAS ID: ebi-a-GCST90018926) included 38,841 cases and 451,248 controls (total *n* = 490,089). Both datasets were based on the GRCh37/hg19 genome build.

The European outcome GWASs combined harmonized phenotype data from UK Biobank and FinnGen. Epilepsy was represented by a broad biobank-based epilepsy phenotype, corresponding to UK Biobank phecode 345.1 and the FinnGen G6_EPLEPSY endpoint. The T2DM phenotype corresponded to UK Biobank phecode 250.2 and the FinnGen E4_DM2 endpoint. Binary-trait GWAS analyses in the source study were conducted using SAIGE and included age, age^2^, sex, age-by-sex interaction, age^2^-by-sex interaction, and the first 20 ancestry principal components as covariates. Detailed information on phenotype definitions, ancestry, sample size, original study, GWAS identifiers, and data sources is provided in Supplementary Table S1.

The circulating-metabolite GWAS included 1091 metabolites and 309 metabolite ratios measured in 8299 participants of European ancestry from the Canadian Longitudinal Study on Aging (CLSA), with summary statistics available through the GWAS Catalog (GCST90199621–GCST90201020).

The HbA1c genetic associations used in constructing the genetically proxied SGLT2-inhibition instrument were obtained from 344,182 unrelated participants of European ancestry in the UK Biobank. Because UK Biobank contributed to both the HbA1c GWAS and the European disease-outcome meta-analyses, partial participant overlap between the exposure-weighting dataset and the outcome datasets cannot be excluded. In two-sample MR, participant overlap may bias estimates toward the corresponding observational association, particularly when instrument strength is limited. The magnitude of the observed association should therefore be interpreted cautiously. The exact degree of overlap could not be quantified from the publicly available summary-level data. In contrast, the metabolomic GWAS was based on the independent CLSA cohort. Possible sample overlap is therefore acknowledged as a limitation of the two-sample MR design.

### 2.5. The Statistical Analysis and Sensitivity Analysis

The associations of genetically proxied SGLT2 inhibition with T2DM and epilepsy were evaluated using two-sample Mendelian randomization (MR). During construction of the exposure instrument, the genetic effect estimates were oriented so that a positive exposure coefficient represented a greater degree of genetically proxied SGLT2 inhibition, rather than a higher HbA1c level. Exposure and outcome summary statistics were subsequently harmonized to the same effect allele before MR estimation. Effect estimates were expressed as odds ratios (ORs) with 95% confidence intervals (CIs), with an OR > 1 indicating higher odds of the outcome and an OR < 1 indicating lower odds. The MR effect estimates correspond to the scale of the genetically proxied exposure used for instrument construction and should not be interpreted as the effect of a 1% or 1 mmol/mol change in HbA1c, nor as the treatment effect of a pharmacological SGLT2 inhibitor. A two-step MR framework was used for exploratory pathway screening to identify circulating metabolites or metabolite ratios that might lie on the pathway between the genetically proxied exposure and epilepsy. For exploratory screening, metabolic traits showing nominal associations with epilepsy were carried forward to the second step and evaluated for association with the genetically proxied exposure. These analyses were intended to prioritize candidate metabolic signals rather than to establish formal causal mediation. In the first step, we estimated the associations of all circulating metabolic traits with epilepsy. Associations with *p* < 0.05 were considered nominally significant, and false discovery rate (FDR) correction was applied to account for multiple testing. For exploratory pathway screening, metabolic traits showing nominal associations with epilepsy were carried forward to the second step as outcomes to estimate their associations with genetically proxied SGLT2 inhibition.

The inverse-variance weighted (IVW) method was used as the primary estimator. MR-Egger regression, weighted-median, simple-mode, and weighted-mode methods were additionally applied to evaluate the consistency of the estimates across alternative MR assumptions.

Potential horizontal pleiotropy was assessed using the MR-Egger intercept test, while the Mendelian Randomization Pleiotropy RESidual Sum and Outlier (MR-PRESSO) procedure was used to identify influential outlying variants. When outliers were detected, the corresponding single-nucleotide polymorphisms (SNPs) were removed and the MR analysis was repeated. Heterogeneity among the SNP-specific estimates was evaluated using Cochran’s Q test, with *p* < 0.05 indicating statistically significant heterogeneity. All statistical analyses were conducted using R software (version 3.4.1). Data harmonization and Mendelian randomization analyses investigating the associations of genetically proxied SGLT2 inhibition with T2DM, epilepsy, and circulating metabolites were performed using the TwoSampleMR package.

## 3. Results

### 3.1. MR Analysis Results of SGLT2 Inhibition on T2DM and Epilepsy

**Figure 2:**
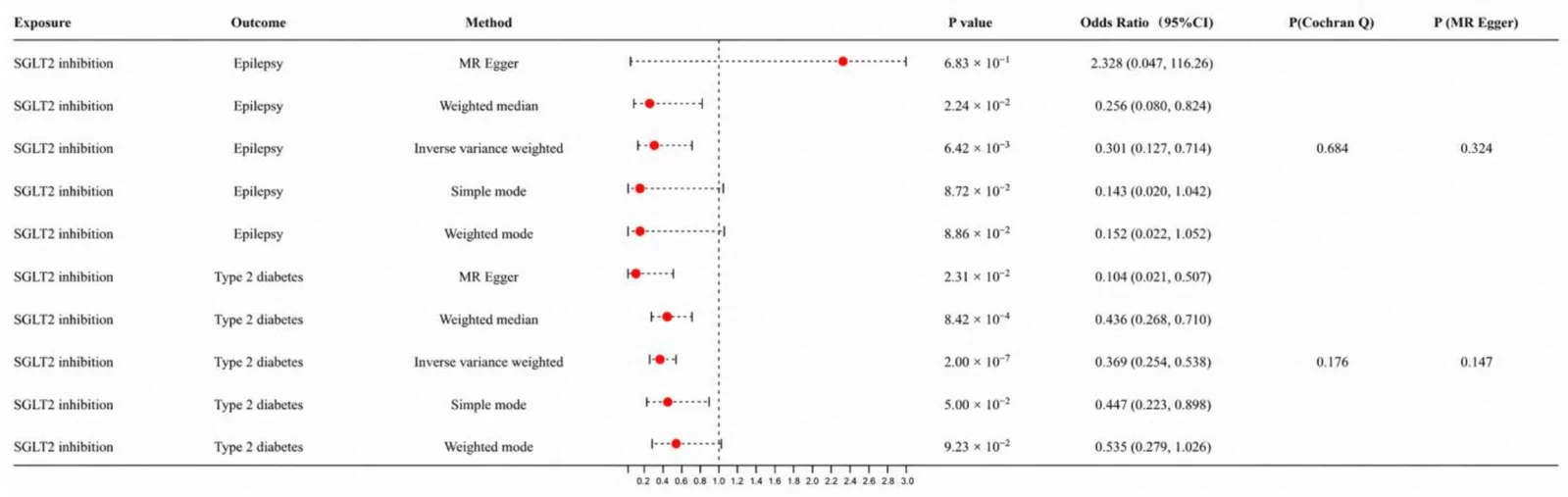
MR analysis results of SGLT2 inhibition on T2DM and epilepsy.

A total of 10 genetic variants were retained in the original instrument set used to proxy SLC5A2-related glycaemic perturbation. The F-statistics for all instruments exceeded the conventional threshold of 10, ranging from 16 to 98, indicating a low likelihood of weak-instrument bias. The positive-control analysis with T2DM showed an association in the expected glycaemic direction, with genetically proxied SGLT2 inhibition associated with lower odds of T2DM [IVW: OR = 0.37, 95% CI: 0.25–0.54; *p* = 2.0 × 10^−7^]. In the primary two-sample MR analysis, genetically proxied SGLT2 inhibition was inversely associated with epilepsy on the genetically proxied exposure scale [IVW: OR = 0.30, 95% CI: 0.13–0.71; *p* = 0.0064]. MR-PRESSO detected no outlier instruments. Sensitivity analyses did not detect statistically significant heterogeneity or directional horizontal pleiotropy; however, these tests have limited power when based on a small number of genetic instruments. For T2DM, the IVW Cochran’s Q test showed no evidence of heterogeneity (Q = 12.71, *p* = 0.176), and the MR-Egger intercept test showed no evidence of directional horizontal pleiotropy (intercept = 0.019, *p* = 0.147). Similarly, for epilepsy, the IVW Cochran’s Q test indicated no significant heterogeneity (Q = 6.55, *p* = 0.684), while the MR-Egger intercept test provided no evidence of directional horizontal pleiotropy (intercept = −0.031, *p* = 0.324). Given the limited number of instruments, non-significant MR-Egger intercept tests should not be interpreted as evidence for the absence of horizontal pleiotropy. Detailed information on the exposure and outcome datasets and the corresponding MR estimates is provided in Figure 2 and Supplementary Tables S1–S3.

### 3.2. Mediation MR of SGLT2 Inhibition, Circulating Metabolites, and Epilepsy Risk

**Figure 3:**
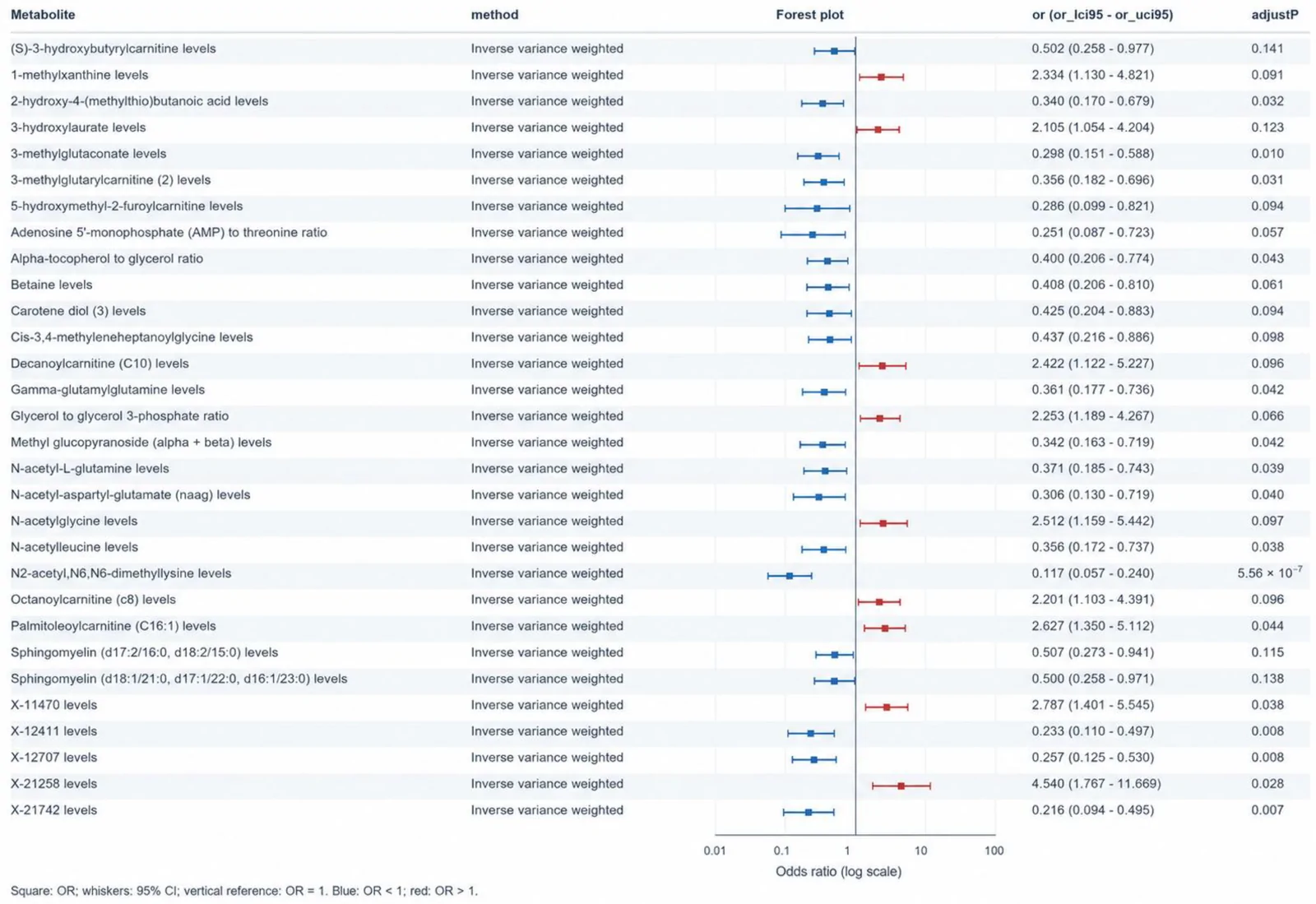
Mendelian randomization associations of circulating metabolites and metabolite ratios with epilepsy risk. Forest plot showing 86 metabolites and 15 metabolite ratios associated with epilepsy at nominal significance (unadjusted *p* < 0.05) using the inverse variance weighted (IVW) method. Squares represent odds ratios (ORs), and horizontal lines indicate 95% confidence intervals (CIs). Blue and red indicate ORs below and above 1, respectively. The vertical reference line denotes OR = 1, and the horizontal axis uses a logarithmic scale. The adjustP column presents false discovery rate (FDR)-adjusted *p* values; only N-acetyltyrosine remained significant after FDR correction (adjusted *p* < 0.05).

**Figure 4:**
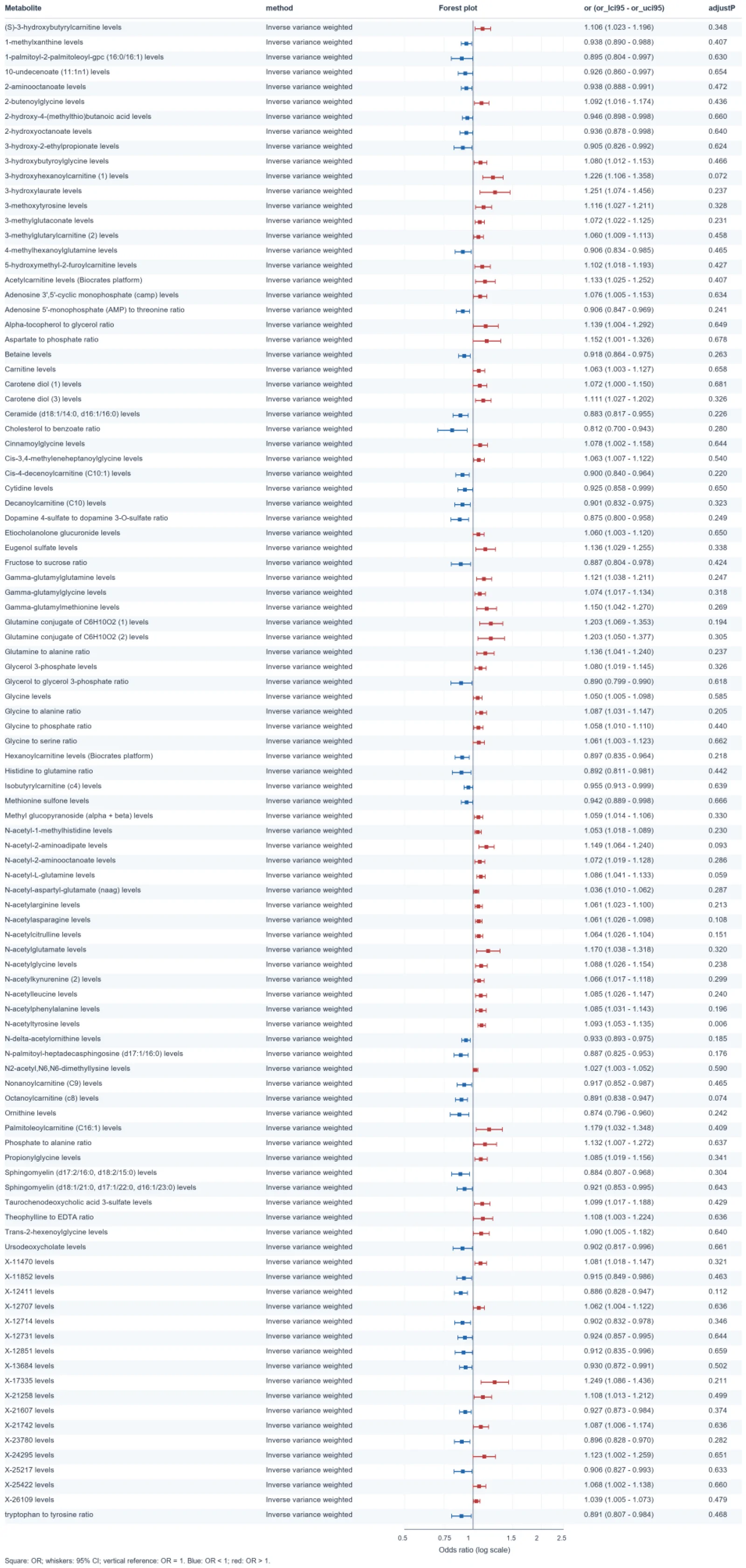
Mendelian randomization associations of genetically proxied SGLT2 inhibition with circulating metabolites and metabolite ratios. Forest plot showing 27 metabolites and three metabolite ratios associated with genetically proxied sodium–glucose cotransporter 2 (SGLT2) inhibition at nominal significance (unadjusted *p* < 0.05) using the inverse variance weighted (IVW) method. Squares represent exponentiated IVW estimates, displayed as ORs, and horizontal lines indicate 95% confidence intervals (CIs). Blue and red indicate estimates below and above 1, respectively. The vertical reference line denotes 1, and the horizontal axis uses a logarithmic scale. The adjustP column presents false discovery rate (FDR)-adjusted *p* values; 15 metabolites and one metabolite ratio remained significant after FDR correction (adjusted *p* < 0.05).

We first evaluated the genetically predicted effects of 1091 circulating metabolites and 309 metabolite ratios on epilepsy risk. At the nominal significance level, 86 metabolites and 15 metabolite ratios showed evidence of associations with epilepsy. After correction for multiple testing using the false discovery rate (FDR), only N-acetyltyrosine remained significantly associated with epilepsy [IVW: OR = 1.093, 95% CI: 1.053–1.135; *p* = 4.0 × 10^−6^; FDR-adjusted *p* = 0.0056]. The complete MR results for these metabolite–epilepsy associations are presented in Figure 3 and Figure 4 and Supplementary Tables S4 and S5.

Next, the 86 metabolites and 15 metabolite ratios showing nominal associations with epilepsy were treated as outcomes to evaluate their associations with genetically proxied SGLT2 inhibition. At nominal significance, genetically proxied SGLT2 inhibition was associated with 27 metabolites and three metabolite ratios. After FDR correction, 15 metabolites and one metabolite ratio remained statistically significant. Among the identified metabolites, genetically proxied SGLT2 inhibition was associated with lower levels of N2-acetyl,N6,N6-dimethyllysine [OR = 0.12, 95% CI: 0.057–0.24; FDR-adjusted *p* = 5.6 × 10^−7^], 3-methylglutaconate [OR = 0.30, 95% CI: 0.15–0.59; FDR-adjusted *p* = 0.0096], 3-methylglutarylcarnitine (2) [OR = 0.36, 95% CI: 0.18–0.70; FDR-adjusted *p* = 0.031], 2-hydroxy-4-(methylthio)butanoic acid [OR = 0.34, 95% CI: 0.17–0.68; FDR-adjusted *p* = 0.032], N-acetylleucine [OR = 0.36, 95% CI: 0.17–0.74; FDR-adjusted *p* = 0.038], N-acetyl-L-glutamine [OR = 0.37, 95% CI: 0.19–0.74; FDR-adjusted *p* = 0.039], N-acetyl-aspartyl-glutamate (NAAG) [OR = 0.31, 95% CI: 0.13–0.72; FDR-adjusted *p* = 0.040], methyl glucopyranoside (alpha + beta) [OR = 0.34, 95% CI: 0.16–0.72; FDR-adjusted *p* = 0.042], gamma-glutamylglutamine [OR = 0.36, 95% CI: 0.18–0.74; FDR-adjusted *p* = 0.042], and the alpha-tocopherol-to-glycerol ratio [OR = 0.40, 95% CI: 0.21–0.77; FDR-adjusted *p* = 0.043]. In contrast, palmitoleoylcarnitine (C16:1) was positively associated with genetically proxied SGLT2 inhibition [OR = 2.63, 95% CI: 1.35–5.11; FDR-adjusted *p* = 0.044]. Five additional signals (X-21742, X-12707, X-12411, X-21258, and X-11470) corresponded to metabolites that have not yet been chemically identified. The corresponding estimates are summarized in Figure 4 and Supplementary Table S3.

Exploratory two-step pathway analysis identified nominal candidate signals involving 3-methylglutaconate (*p* = 0.027) and N-acetylleucine (*p* = 0.046). However, neither candidate signal remained statistically significant after Benjamini–Hochberg correction for multiple testing across the nine candidate traits (FDR-adjusted *p* ≈ 0.095 for both). Moreover, neither metabolite survived FDR correction in the preceding metabolite-to-epilepsy analysis. These findings should therefore be regarded as exploratory signals rather than statistically established mediation effects.

## 4. Discussion

Exploratory two-step MR analyses identified nominal candidate metabolic signals involving 3-methylglutaconate and N-acetylleucine. However, these associations did not withstand correction for multiple testing and therefore do not establish a mediating role. They should instead be considered hypothesis-generating metabolic signals for future replication and mechanistic investigation.

Experimental studies have reported antioxidant and anti-inflammatory effects of pharmacological SGLT2 inhibition, including reduced oxidative stress and modulation of inflammatory signaling [23,24,25,26]. Neuroprotective effects involving neuroinflammation, cerebral energy metabolism, and related pathways have also been proposed [27]. Epilepsy is characterized by recurrent abnormal neuronal activity [28], and neuroinflammatory signaling has been implicated in epileptogenesis [29,30,31]. IL-1β and NLRP3-related signaling are among the inflammatory pathways implicated in epilepsy [32]. Experimental studies further suggest that pharmacological SGLT2 inhibition can modulate NLRP3-related neuroinflammatory signaling [33]. However, the present MR analysis did not directly assess oxidative stress, cytokines, neuroinflammation, or NLRP3 activity; these mechanisms should therefore be regarded as biologically plausible hypotheses rather than mechanisms established by our findings.

Multiple studies support the use of circulating metabolites as candidate biomarkers of neurological disorders. Because biomarker concentrations vary with biological or pathological states, they may assist with diagnosis, prognosis, and treatment monitoring [34]. Among the metabolic traits associated with genetically proxied SGLT2 inhibition, we identified several pathways that could plausibly contribute to epilepsy.

Direct evidence linking the circulating analyte annotated as N-acetylleucine to epilepsy is currently lacking. Importantly, this metabolomic annotation should not be assumed to be stereochemically identical to pharmacologically administered N-acetyl-L-leucine unless the analytical platform specifically resolves the L-enantiomer [35]. Accordingly, mechanistic observations reported for pharmacological N-acetyl-L-leucine cannot be directly extrapolated to the circulating N-acetylleucine signal identified in the present analysis. Glutamatergic and GABAergic neurotransmission are central to neuronal excitability [36,37,38], and disruption of the glutamate–GABA balance has been implicated in seizure generation and epilepsy [38]. However, our analyses did not measure neurotransmitter metabolism, and no mechanistic link between circulating N-acetylleucine and this pathway can be inferred from the present data.

No study has directly established a role for 3-methylglutaconate in epileptogenesis. Nevertheless, elevated urinary 3-methylglutaconate has been reported among the biochemical abnormalities observed in some patients with ECHS1 deficiency, a mitochondrial disorder in which seizures may occur [39,40]. These observations establish only a clinical co-occurrence and do not imply that 3-methylglutaconate itself contributes causally to epileptogenesis [41]. The pathways through which this metabolite might influence seizure onset remain unknown and require experimental evaluation.

Clarifying these pathways could reveal additional therapeutic opportunities for epilepsy. At present, however, it is uncertain whether changes in circulating metabolites account for any antiseizure effect of SGLT2 inhibition. Our findings therefore define a hypothesis for subsequent mechanistic and clinical investigation rather than establishing a treatment strategy.

Several limitations should be considered. First, the contributing GWAS datasets predominantly included participants of European ancestry, which may limit the generalizability of our findings to other populations. Second, despite broad coverage of the circulating metabolome, unavailable pyrimidine GWAS data prevented evaluation of this metabolic domain [42]. In addition, because SLC5A2 has a major physiological role in renal proximal tubular glucose reabsorption, eQTL information derived from non-renal tissues may not fully capture kidney-specific SLC5A2 regulation. The original genetic instrument set was also not restricted exclusively to variants located within the SLC5A2 locus; therefore, target specificity cannot be assumed for every retained variant. Accordingly, the tissue specificity and locus specificity of the instrument set should be regarded as limitations in target interpretation. Moreover, partial participant overlap between the UK Biobank-derived HbA1c dataset and the disease-outcome GWASs cannot be excluded, although the exact degree of overlap could not be quantified from the available summary-level data. Reverse MR from epilepsy to the candidate metabolites was not performed; therefore, reverse directionality in the metabolite–epilepsy associations cannot be excluded. Accordingly, the direction of the candidate metabolite–epilepsy relationships remains unresolved, and these findings should be interpreted only as hypothesis-generating. Furthermore, drug-target MR estimates reflect lifelong genetically proxied perturbation of SLC5A2-related biology and should not be interpreted as directly equivalent to pharmacological SGLT2 inhibitor treatment. Differences in exposure magnitude, duration, dose, tissue specificity, and potential off-target effects should therefore be considered when translating these genetic findings to clinical pharmacotherapy. The mediation findings should also be interpreted cautiously, as the nominal indirect effects observed for 3-methylglutaconate and N-acetylleucine did not remain statistically significant after correction for multiple testing and therefore should be regarded as exploratory. Another important limitation concerns the definition and heterogeneity of the epilepsy outcome. The primary epilepsy GWAS was based on a broad biobank-derived phenotype comprising 4382 cases and lacked detailed clinical subclassification of epilepsy syndromes. The available summary-level information also does not allow us to determine whether acute symptomatic seizures or isolated seizure codes were uniformly excluded across the contributing cohorts. Accordingly, the OR of 0.30 should not be interpreted as indicating a 70% reduction in epilepsy risk achievable with clinical SGLT2 inhibitor treatment. Moreover, focal epilepsy, genetic generalized epilepsy, and epilepsies of different etiologies have distinct genetic architectures; therefore, an association identified using a broad epilepsy phenotype may not generalize uniformly across epilepsy subtypes. Age at epilepsy onset was also unavailable in the summary-level outcome data, precluding separate evaluation of late-onset epilepsy. The magnitude of the epilepsy IVW estimate should also be interpreted cautiously. The epilepsy point estimate was larger in magnitude than that observed for the T2DM positive-control analysis, whereas the MR-Egger point estimate was directionally discordant with the IVW estimate. Together with the limited number of instruments and the relatively small number of epilepsy cases, this discrepancy indicates substantial statistical uncertainty and possible instability of the epilepsy estimate. Accordingly, the IVW estimate should not be interpreted as evidence that the neurological effect of SGLT2-related perturbation is greater than its established glycaemic effect. Replication in larger, deeply phenotyped datasets, such as the 2023 ILAE Consortium GWAS with subtype-specific analyses, will be important to determine whether the association differs across epilepsy subtypes. In addition, because only 10 genetic instruments were available, the sensitivity analyses had limited power to detect horizontal pleiotropy or other violations of the instrumental-variable assumptions, and residual pleiotropy, reverse directionality, or confounding cannot be completely excluded. Finally, although experimental observations are directionally consistent with our estimates, clinical confirmation remains lacking [12].

## 5. Conclusions

Our findings suggest that genetically proxied SGLT2 inhibition is associated with lower odds of epilepsy. Exploratory two-step analyses identified 3-methylglutaconate and N-acetylleucine as candidate metabolic signals of interest; however, neither signal remained statistically significant after correction for multiple testing and they should not be interpreted as established mediators. Although previous experimental studies have implicated antioxidant and anti-inflammatory pathways in the neuroprotective effects of pharmacological SGLT2 inhibition, the present genetic analyses do not directly establish these mechanisms. These metabolic and mechanistic findings should therefore be considered hypothesis-generating and require further validation in independent cohorts and experimental studies.

## Data Availability

All data generated or analyzed during this study are included in this published article and its supplementary information files. The analyses were performed in R (4.3.1) using the TwoSampleMR (0.6.8) and MR-PRESSO (1.0) packages. The original complete analysis scripts are no longer available. Key analytical parameters, harmonized variant-level data, and sufficient methodological details to reproduce the primary MR analysis are provided in the Methods and Supplementary Materials.

## Supplementary Materials

The following supporting information can be downloaded at: https://www.mdpi.com/article/10.3390/biomedicines14102406/s1, Table S1: Dataset source and description.; Table S2: Genetic variants selected as instrumental variables for genetically proxied SGLT2 inhibition.; Table S3: The result of mendelian randomization analysis; Table S4: The effects of circulating metabolites on Epilepsy.; Table S5: The effects of SGLT2 inhibitors on circulating metabolites.; File S1: STROBE-MR checklist.
