# Supplementary material for: Genetically Proxied SGLT2 Inhibition and Risk of Epilepsy: A Mediation Mendelian Randomization Study of Circulating Metabolites
Source: Biomedicines. 2026 Oct 21;14(10):2406. doi: 10.3390/biomedicines14102406 (PMC13702378; doi:10.3390/biomedicines14102406)
Supplement: Supplementary file 1 [file biomedicines-14-02406-s001.zip › File S1 STROBE-MR checklist.pdf]

STROBE-MR checklist of recommended items to address in reports of Mendelian randomization studies[1 2]

Item No.	Section		Checklist item	Page No.	Relevant text from manuscript
1	**TITLE and ABSTRACT**		Indicate Mendelian randomization (MR) as the study's design in the title and/or the abstract if that is a main purpose of the study	Title/Abstract not included in the provided revised manuscript file	The revised manuscript file provided for checklist verification begins with the Introduction. Please verify this item against the final submission title/abstract; no new title/abstract wording has been inferred.
	**INTRODUCTION**				
2	**Background**		Explain the scientific background and rationale for the reported study. What is the exposure? Is a potential causal relationship between exposure and outcome plausible? Justify why MR is a helpful method to address the study question	1–2	The Introduction describes epilepsy burden and unmet treatment needs, summarizes experimental and clinical evidence concerning SGLT2 inhibition, and explains the rationale for using MR to investigate genetically proxied SGLT2 inhibition, circulating metabolites, and epilepsy.
3	**Objectives**		State specific objectives clearly, including pre-specified causal hypotheses (if any). State that MR is a method that, under specific assumptions, intends to estimate causal effects	2	"Mendelian randomization (MR) uses inherited genetic variants as instrumental variables to estimate associations that may inform causal inference under specific assumptions." The study then states that two-sample MR and two-step MR were used to investigate genetically proxied SGLT2 inhibition, epilepsy, and circulating metabolic traits.
	**METHODS**				
4	**Study design and data sources**		Present key elements of the study design early in the article. Consider including a table listing sources of data for all phases of the study. For each data source contributing to the analysis, describe the following:		
		a)	Setting: Describe the study design and the underlying population, if possible. Describe the setting, locations, and relevant dates, including periods of recruitment, exposure, follow-up, and data collection, when available.	2–4; Supplementary Table 1	Sections 2.1–2.4 describe the two-sample/two-step MR design and the contributing GWAS resources: UK Biobank HbA1c data, GTEx/eQTLGen SLC5A2 expression resources, UK Biobank + FinnGen outcome GWASs, and

					the CLSA metabolomics GWAS. Supplementary Table 1 summarizes dataset sources and characteristics.
		b)	Participants: Give the eligibility criteria, and the sources and methods of selection of participants. Report the sample size, and whether any power or sample size calculations were carried out prior to the main analysis	3–4; Supplementary Table 1	HbA1c: 344,182 unrelated participants of European ancestry from UK Biobank. Epilepsy: 4,382 cases and 453,928 controls (n=458,310). T2DM: 38,841 cases and 451,248 controls (n=490,089). Metabolomics: 8,299 CLSA participants of European ancestry.
		c)	Describe measurement, quality control and selection of genetic variants	2–3; Supplementary Table 2	Section 2.2 describes SLC5A2-expression/HbA1c instrument selection, colocalization (posterior probability >70%), and LD clumping using the 1000 Genomes European reference panel (r^2<0.001 within 10,000 kb). Supplementary Table 2 reports the retained instrument-level characteristics.
		d)	For each exposure, outcome, and other relevant variables, describe methods of assessment and diagnostic criteria for diseases	3–4; Supplementary Table 1	Section 2.4 reports the epilepsy and T2DM phenotype definitions and data sources: epilepsy corresponded to UK Biobank phecode 345.1 and FinnGen G6_EPLEPSY; T2DM corresponded to UK Biobank phecode 250.2 and FinnGen E4_DM2. Sample sizes, ancestry, genome build, and source-study covariates are also reported.
		e)	Provide details of ethics committee approval and participant informed consent, if relevant	10	Ethics approval: “We confirm that we have read the Journal’s position on issues involved in ethical publication and affirm that this report is consistent with those guidelines.” The analyses use previously collected summary-level GWAS data.
5	**Assumptions**		Explicitly state the three core IV assumptions for the main analysis (relevance, independence and exclusion restriction) as well assumptions for any additional or sensitivity analysis	2–5	The three core IV assumptions are not listed verbatim as a three-item statement in the current manuscript. Relevance is addressed

					through SLC5A2/HbA1c instrument selection and F-statistics; independence/exclusion-restriction concerns are evaluated indirectly through LD clumping, MR-Egger, MR-PRESSO, and heterogeneity analyses.
6	**Statistical methods: main analysis**		Describe statistical methods and statistics used		
		a)	Describe how quantitative variables were handled in the analyses (i.e., scale, units, model)	4	Effect estimates are reported as ORs with 95% CIs. The manuscript states that estimates correspond to the genetically proxied exposure scale and should not be interpreted as a 1% or 1 mmol/mol HbA1c change or as a pharmacological SGLT2 inhibitor treatment effect.
		b)	Describe how genetic variants were handled in the analyses and, if applicable, how their weights were selected	2–3; Supplementary Table 2	Genetic variants were selected using SLC5A2 expression and HbA1c associations, colocalization, and stringent LD clumping (r^2<0.001, 10,000-kb window; European 1000 Genomes reference). Instrument-level exposure/outcome associations and F-statistics are reported in Supplementary Table 2.
		c)	Describe the MR estimator (e.g. two-stage least squares, Wald ratio) and related statistics. Detail the included covariates and, in case of two-sample MR, whether the same covariate set was used for adjustment in the two samples	3–5	IVW was the primary MR estimator; MR-Egger, weighted-median, simple-mode, and weighted-mode methods were used as complementary estimators. MR-Egger intercept, MR-PRESSO, and Cochran's Q were used for sensitivity analyses. Source-study GWAS covariates for the disease outcomes are reported in Section 2.4.
		d)	Explain how missing data were addressed	Not reported	Participant-level missing-data handling is not reported because the study uses published summary-level GWAS data; no individual-level imputation or missing-data analysis was performed in the reported MR workflow.

		e) If applicable, indicate how multiple testing was addressed	4–6	FDR correction was applied to metabolome-wide analyses. Candidate mediation signals were additionally evaluated using Benjamini–Hochberg correction across the nine candidate mediators; neither 3-methylglutaconate nor N-acetylleucine remained significant after correction (FDR-adjusted P≈0.095).
7	**Assessment of assumptions**	Describe any methods or prior knowledge used to assess the assumptions or justify their validity	2–5; Supplementary Tables 2–3	Instrument relevance was evaluated through association thresholds and F-statistics; LD clumping minimized correlation. MR-Egger intercept, MR-PRESSO, and Cochran's Q were used to assess pleiotropy/outliers/heterogeneity. The manuscript cautions that these tests have limited power with only 10 instruments.
8	**Sensitivity analyses and additional analyses**	Describe any sensitivity analyses or additional analyses performed (e.g. comparison of effect estimates from different approaches, independent replication, bias analytic techniques, validation of instruments, simulations)	4–5; Supplementary Table 3	Sensitivity analyses included MR-Egger regression, weighted-median, simple-mode, weighted-mode, MR-PRESSO, and Cochran's Q. MR-PRESSO detected no outlier instruments; heterogeneity and MR-Egger intercept results are reported for T2DM and epilepsy.
9	**Software and pre-registration**			
		a) Name statistical software and package(s), including version and settings used	5	All statistical analyses were conducted using R software (version 3.4.1). Data harmonization and MR analyses of genetically proxied SGLT2 inhibition with T2DM, epilepsy, and circulating metabolites were performed using the TwoSampleMR package; MR-PRESSO was used for outlier/pleiotropy assessment.
		b) State whether the study protocol and details were pre-registered (as well as when and where)	Not reported	No study pre-registration is reported in the current manuscript.
	**RESULTS**			
10	**Descriptive data**			
		a) Report the numbers of individuals at each stage of included studies and reasons for exclusion. Consider use of a flow diagram	3–4; Supplementary Table 1; Figure 1	Dataset sample sizes are reported in Section 2.4 and Supplementary Table 1: HbA1c n=344,182; epilepsy 4,382 cases/453,928 controls; T2DM 38,841 cases/451,248 controls; metabolomics n=8,299. Figure 1 summarizes the study workflow.

		b)	Report summary statistics for phenotypic exposure(s), outcome(s), and other relevant variables (e.g. means, SDs, proportions)	3–4; Supplementary Tables 1–2	Summary characteristics of the contributing GWAS datasets are provided in Supplementary Table 1; instrument-level SNP information, including exposure/outcome associations and allele frequencies, is provided in Supplementary Table 2.
		c)	If the data sources include meta-analyses of previous studies, provide the assessments of heterogeneity across these studies	Not reported	The epilepsy and T2DM outcome GWASs are European-ancestry meta-analyses of UK Biobank and FinnGen data, but between-cohort heterogeneity statistics from the source meta-analyses are not reproduced in the current manuscript.
		d)	For two-sample MR:<br>i. Provide justification of the similarity of the genetic variant-exposure associations between the exposure and outcome samples<br>ii. Provide information on the number of individuals who overlap between the exposure and outcome studies	4; 8	The manuscript reports that the HbA1c weighting dataset was derived from UK Biobank and that UK Biobank also contributed to the disease-outcome meta-analyses; therefore, partial participant overlap cannot be excluded and the exact degree of overlap cannot be quantified from public summary data. This is explicitly discussed as a limitation.
11	**Main results**				
		a)	Report the associations between genetic variant and exposure, and between genetic variant and outcome, preferably on an interpretable scale	Supplementary Table 2	Supplementary Table 2 reports the 10 retained genetic instruments, including effect/other alleles, exposure and epilepsy effect estimates and SEs, allele frequencies, instrument-retention information, variance explained, and F-statistics.
		b)	Report MR estimates of the relationship between exposure and outcome, and the measures of uncertainty from the MR analysis, on an interpretable scale, such as odds ratio or relative risk per SD difference	5–6; Figures 2–4; Supplementary Tables 3–5	The primary IVW estimate for epilepsy was OR=0.30 (95% CI 0.13–0.71; P=0.0064) on the genetically proxied exposure scale. T2DM positive-control and metabolomic MR estimates, uncertainty, nominal P values, and FDR-adjusted results are reported in the Results, figures, and Supplementary Tables.

		c)	If relevant, consider translating estimates of relative risk into absolute risk for a meaningful time period	Not applicable	Absolute-risk translation was not performed because the MR estimate is on a genetically proxied exposure scale and is not directly interpretable as a pharmacological treatment effect over a defined clinical time horizon.
		d)	Consider plots to visualize results (e.g. forest plot, scatterplot of associations between genetic variants and outcome versus between genetic variants and exposure)	Figures 1–4; Supplementary Tables 3–5	Figure 1 presents the study design; Figure 2 presents the primary T2DM/epilepsy MR estimates; Figures 3–4 provide high-dimensional metabolomic overviews. Exact effect estimates, 95% CIs, P values, and FDR-adjusted P values are provided in the Supplementary Tables.
12	**Assessment of assumptions**				
		a)	Report the assessment of the validity of the assumptions	2–5; Supplementary Tables 2–3	Instrument relevance and strength were evaluated through selection criteria and F-statistics (16–98). LD clumping was applied. Pleiotropy/outliers/heterogeneity were assessed using MR-Egger intercept, MR-PRESSO, and Cochran's Q, with explicit caution regarding limited power from only 10 instruments.
		b)	Report any additional statistics (e.g., assessments of heterogeneity across genetic variants, such as I^2, Q statistic or E-value)	5; Supplementary Table 3	Cochran's Q and MR-Egger intercept results are reported. T2DM: Q=12.71, P=0.176; MR-Egger intercept=0.019, P=0.147. Epilepsy: Q=6.55, P=0.684; MR-Egger intercept=−0.031, P=0.324.
13	**Sensitivity analyses and additional analyses**				
		a)	Report any sensitivity analyses to assess the robustness of the main results to violations of the assumptions	5; Supplementary Table 3	Sensitivity analyses included MR-Egger, weighted-median, simple-mode, weighted-mode, MR-PRESSO, and Cochran's Q. The manuscript explicitly states that non-significant MR-Egger tests should not be interpreted as proof that horizontal pleiotropy is absent.
		b)	Report results from other sensitivity analyses or additional analyses	5–6	Additional analyses included the T2DM positive-control analysis and exploratory two-step metabolomic MR with FDR/Benjamini–Hochberg correction. No established mediation effect was claimed after multiple-testing correction.

		c)	Report any assessment of direction of causal relationship (e.g., bidirectional MR)	8	Reverse MR from epilepsy to candidate metabolites was not performed; the manuscript explicitly states that reverse directionality in metabolite–epilepsy associations cannot be excluded.
		d)	When relevant, report and compare with estimates from non-MR analyses	Not applicable	No formal non-MR effect estimate was directly compared with the MR estimate. External experimental and clinical literature is discussed qualitatively in the Introduction and Discussion.
		e)	Consider additional plots to visualize results (e.g., leave-one-out analyses)	Not performed	Leave-one-out plots were not performed/reported. The manuscript instead reports multiple complementary MR estimators, MR-PRESSO, MR-Egger intercept, and Cochran’s Q, and acknowledges the limited sensitivity-analysis power with 10 instruments.
	**DISCUSSION**				
14	**Key results**		Summarize key results with reference to study objectives	5–6; 9	The Results and Discussion report an inverse association between genetically proxied SGLT2 inhibition and epilepsy, while the two-step metabolomic findings are explicitly described as exploratory because the candidate indirect-effect signals did not survive multiple-testing correction. The Conclusion reiterates this cautious interpretation.
15	**Limitations**		Discuss limitations of the study, taking into account the validity of the IV assumptions, other sources of potential bias, and imprecision. Discuss both direction and magnitude of any potential bias and any efforts to address them	8–9	The Limitations section discusses European ancestry, incomplete metabolomic coverage, non-renal eQTL relevance, possible UK Biobank sample overlap, reverse directionality, genetic-proxy versus pharmacological-treatment differences, multiple testing, broad/heterogeneous epilepsy phenotype, unavailable age at onset, limited sensitivity-analysis power, and residual pleiotropy/confounding.
16	**Interpretation**				
		a)	Meaning: Give a cautious overall interpretation of results in the context of their limitations and in comparison with other studies	6–9	The Discussion interprets the findings cautiously and distinguishes hypothesis-generating metabolomic signals from established mediation. The Conclusion states that the metabolic and mechanistic findings require

				validation in independent cohorts and experimental studies.
		b) Mechanism: Discuss underlying biological mechanisms that could drive a potential causal relationship between the investigated exposure and the outcome, and whether the gene-environment equivalence assumption is reasonable. Use causal language carefully, clarifying that IV estimates may provide causal effects only under certain assumptions	6–8	The Discussion reviews antioxidant, anti-inflammatory, NLRP3, glutamatergic/GABAergic, N-acetylleucine, and 3-methylglutaconate hypotheses while explicitly stating that these mechanisms were not established by the present MR analysis. The Limitations section explains that lifelong genetic perturbation is not equivalent to pharmacological SGLT2 inhibition.
		c) Clinical relevance: Discuss whether the results have clinical or public policy relevance, and to what extent they inform effect sizes of possible interventions	8–9	The manuscript states that genetically proxied effects should not be interpreted as clinical SGLT2 inhibitor treatment effects and specifically cautions that OR=0.30 should not be interpreted as a 70% treatment-associated reduction in epilepsy risk. Clinical confirmation remains lacking.
17	**Generalizability**	Discuss the generalizability of the study results (a) to other populations, (b) across other exposure periods/timings, and (c) across other levels of exposure	8–9	Generalizability limitations are explicitly discussed: predominantly European ancestry; broad epilepsy phenotype; unavailable age at onset; potential subtype differences; and differences in magnitude, duration, dose, tissue specificity, and off-target effects between lifelong genetic perturbation and pharmacological treatment.
	**OTHER INFORMATION**			
18	**Funding**	Describe sources of funding and the role of funders in the present study and, if applicable, sources of funding for the databases and original study or studies on which the present study is based	10	“The preparation of this article was supported by the National Natural Science Foundation of China (No 82201445); the National Natural Science Foundation of China (Grant No.82171309).”
19	**Data and data sharing**	Provide the data used to perform all analyses or report where and how the data can be accessed, and reference these sources in the article. Provide the statistical code needed to reproduce the results in the article, or report whether the code is publicly accessible and if so, where	10; Supplementary Materials	The manuscript states that all data generated or analyzed are included in the article and supplementary files. The Code Availability statement reports that analyses were performed in R using TwoSampleMR and MR-PRESSO; the original complete scripts are no longer available, while key analytical parameters and harmonized variant-level data are provided in the Methods and Supplementary Materials.
20	**Conflicts of Interest**	All authors should declare all potential conflicts of interest	10	“The authors declare that they have no competing interests.”

This checklist is copyrighted by the Equator Network under the Creative Commons Attribution 3.0 Unported (CC BY 3.0) license.

1. Skrivankova, V.W.; Richmond, R.C.; Woolf, B.A.R.; Yarmolinsky, J.; Davies, N.M.; Swanson, S.A.; VanderWeele, T.J.; Higgins, J.P.T.; Timpson, N.J.; Dimou, N.; et al. Strengthening the Reporting of Observational Studies in Epidemiology Using Mendelian Randomization: The STROBE-MR Statement. *JAMA* **2021**, *326*, 1614–1621. https://doi.org/10.1001/jama.2021.18236.
2. Skrivankova, V.W.; Richmond, R.C.; Woolf, B.A.R.; Davies, N.M.; Swanson, S.A.; VanderWeele, T.J.; Higgins, J.P.; Timpson, N.J.; Dimou, N.; Langenberg, C. Strengthening the Reporting of Observational Studies in Epidemiology using Mendelian Randomisation (STROBE-MR): Explanation and Elaboration. *BMJ* **2021**, *375*, n2233.
